# Overexpression of WRINKLED1 improves the weight and oil content in seeds of flax (*Linum usitatissimum* L.)

**DOI:** 10.3389/fpls.2022.1003758

**Published:** 2022-09-30

**Authors:** Wenjuan Li, Limin Wang, Yanni Qi, Yaping Xie, Wei Zhao, Zhao Dang, Jianping Zhang

**Affiliations:** Institute of Crop Research, Gansu Academy of Agricultural Sciences, Lanzhou, China

**Keywords:** *Linum usitatissimum.* L, WRINKLED1, oil content, gene expression, seed weight

## Abstract

Seeds of flax (*Linum usitatissimum* L.) are highly rich in both oil and linolenic acid (LIN). It is crucial for flax agricultural production to identify positive regulators of fatty acid biosynthesis. In this study, we find that WRINKLED1 transcription factors play important positive roles during flax seed oil accumulation. Two WRINKLED1 genes, *LuWRI1a* and *LuWRI1b*, were cloned from flax, and *LuWRI1a* was found be expressed predominantly in developing seeds during maturation. Overexpression of *LuWRI1a* increased seed size, weight, and oil content in Arabidopsis and increased seed storage oil content in transgenic flax without affecting seed production or seed oil quality. The rise in oil content in transgenic flax seeds was primarily attributable to the increase in seed weight, according to a correlational analysis. Furthermore, overexpression or interference of *LuWRI1a* upregulated the expression of genes in the fatty acid biosynthesis pathway and LAFL genes, and the expression level of WRI1 was highly significantly positively associated between L1L, LEC1, and BCCP2. Our findings give a theoretical scientific foundation for the future application of genetic engineering to enhance the oil content of plant seeds.

## Introduction

Plant seeds can store a large amount of oils as triacylglycerol (TAG), serving as energy and carbon reserves for seed germination. TAG is an important industrial raw material for the production of detergents and lubricants, in addition to being essential to human and animal nutrition (Cahoon et al., 2007; Durrett et al., 2008; Dyer et al., 2008). Widely cultivated in India, Canada, and China, flax (*Linum usitatissimum* L.) is a crop with dual uses for oil and fiber (Huis et al., 2010). As an oil crop, flax is famous for its high contents of oil and unsaturated fatty acids in seeds, which generally contains 40%–50% of oil (Green, 1986); around 73% of polyunsaturated fatty acids and approximately 50% of the fatty acids are α-linolenic acid (C18:3, ALA) (Daun and Declercq, 1994).

TAG biosynthesis is composed of two primary processes, fatty acid biosynthesis and TAG assembly. Numerous genes are involved, such as CCP2 subunit (BCCP2), fatty acyl-ACP thioesterase (FATA/FATB), diacylglycerol acyltransferase (DGAT), and phosphatidylcholine: diacylglycerol acyltransferase (PDAT) (Zou et al., 1999; Dahlqvist et al., 2000). Nonetheless, the regulating mechanisms of oil biosynthesis in plants are not fully understood. Research on bioengineering oil crops with higher oil content require an understanding of the regulation of TAG biosynthesis. According to a number of studies, certain transcription factors (TFs) control the expression of various genes in oil biosynthesis in order to enhance oil yield. For example, the B3 domain TF family includes genes LEAFY COTYLEDON2(LEC2), ABSCISIC ACID INSENSITIVE3 (ABI3), and FUSCA3 (FUS3), in addition to the NF-YB TFs LEC1 and LEC1-like (L1L) that regulate oil biosynthesis *via* the TF wrinkled1 (WRI1) (Parcy et al., 1997; Lotan et al., 1998; To et al., 2006; Boulard et al., 2017; Huang et al., 2022). Collectively, The five TFs discussed above are known as LAFL proteins. Storage proteins and fats are accumulated during seed maturity, and this process is regulated by LAFL processes (Yamamoto et al., 2009; Roscoe et al., 2015).

The *WRI1* gene was initially identified in the Arabidopsis *wri1-1* mutant, where it was given the name *WRINKLED1* due to the epidermal wrinkles in the mutant. In a *wri1*-deficient Arabidopsis mutant, the normal seed surface phenotype can be rescued by overexpressing *AtWRI1*. Compared with the wild type, the mutant is incapable of converting glucose and sucrose into the precursors of fatty acid synthesis throughout seed development, and the activity of numerous glycolytic enzymes, including hexokinase and phosphofructokinase, is decreased, leading to an 80% decrease in oil content of the mutant seeds (Focks and Benning, 1998). WRI1 belongs to the APETALA2 (AP2) TF family (Cernac and Benning, 2004; Masaki et al., 2005), containing two AP2s, including a 14-3-3 and E3 ligase adaptor (BPM) binding motifs, a functional motif of “VYL” (Ma et al., 2013), and a T70 phosphorylation residues. The conserved structure of other WRI1 proteins is the same as in AtWRI1 (Ma et al., 2013; Ma et al., 2015; Yang et al., 2015; An et al., 2017; Kong and Ma, 2018; Ye et al., 2018; Kong et al., 2019; Snell et al., 2019; Tang et al., 2019; Chen et al., 2020).

Several WRI1 orthologs have been identified in plants, including *Glycine max* (Manan et al., 2017; Zhang et al., 2017; Chen et al., 2020), *Oryza sativa* (Mano et al., 2019), *Zea mays* (Shen et al., 2010; Pouvreau et al., 2011), *Brassica napus* (Liu et al., 2010), *Arachis hypogaea* (Tang et al., 2018), *Ricinus communis* (Kim et al., 2013; Ji et al., 2018; Yang et al., 2019), and *Cocos nucifera* (Sun et al., 2017). Previous studies have shown that *AtWRI1* or *WRI1* orthologs significantly elevated seed oil content in transgenic plants (Cernac and Benning, 2004; Liu et al., 2010; Shen et al., 2010; An and Suh, 2015; Yang et al., 2015; Sun et al., 2017; Ye et al., 2018). Moreover, alteration of transcription factor expression may have unfavorable pleiotropic implications on agronomic performance. It is also necessary to determine whether oil increase affects oil quality or yield.

WRI have multiple functions in various plant species; although the multiple roles of *WRI1* genes in various plant species have been identified, the function of its paralog in flax (*Linum usitatissimum* L.) is not yet known. In the study, two *WRINKLED 1* (*WRI1*) genes, *LuWRI1a* and *LuWRI1b*, were found in flax that are strongly expressed in developing seeds throughout seed maturation. We found that overexpressing *LuWRI1a* increased seed oil yield without compromising oil quality or seed yield. The results demonstrated the possible application of transcription factors for enhancing oil production in oil crops.

## Materials and methods

### Plant materials

The experiment utilized *Arabidopsis thaliana* Col-2 as the wild type. The mutant of *Arabidopsis thaliana wri1-1* was obtained from Christoph Benning (East Lansing, Michigan, USA). The seeds were pre-incubated for 3 days at a temperature of 4°C in complete darkness. The flax cultivar Longya 10 (*Linum usitatissimum* cultivar Longya 10) was used in the experiment. Plants were cultivated in a culture room with a day/night temperature range of 22/20°C, a 16-h light/8-h dark (100–150 μmol m^−2^ s^−1^), and 75% relative humidity. Plant materials were harvested at the preset time and rinsed with distilled water, and then the intact root, leaf, stem, flower, and developing boll tissues were sampled using sterilized scissors. All samples were each collected from more than 10 individual plants. Flowers were tagged at anthesis, and some open flowers were gathered on the same day to constitute the floral stage sample set. Medium parts of stems were collected. Developing bolls were taken on 10, 20, 30, 40, and 50 days after anthesis (DAA) for the blooming and green capsule stages, for a total of six timepoints (including flower stage). Tissues were at once frozen in liquid nitrogen and kept at -80°C until RNA was extracted.

### RNA extraction, quality control, and first-strand cDNA synthesis

The RNeasy Plant Mini Kit (Qiagen) was used to extract total RNA in accordance with the manufacturer′s guidelines. The samples showed a 260/280-nm ratio ranging between 1.8 and 2.0 when the RNA concentration was measured using NanoDrop 2000. Using ethidium bromide-stained agarose gels, the purity of RNA was evaluated, and no deterioration was found. The RNA extractions were performed in two biological replicates. Utilizing a PrimeScript II First Strand cDNA Synthesis Kit (TaKaRa) and 2 μg of total RNA, first-strand cDNA was produced. The synthesized cDNA samples were kept at -20°C. Unless otherwise specified, all standard chemicals and organic solvents were purchased from Invitrogen or Takara.

### Cloning *LuWRI1a* and *LuWRI1b* genes from flax

By blasting the NCBI EST database with *AtWRI1* cDNA, eight probable *LuWRI1*-coding regions were detected. This primer was prepared in accordance with the manufacturer′s guidelines for 3′RACE and 5′RACE analyses. We used a 5′-RACE kit to get the 5′ end (Takara, Madison, WI, USA). The 3′ end was obtained by 3′-RACE using a kit. Flax *LuWRI1* was found by scanning the flax genomic database with the BLAST algorithm and the sequence of Arabidopsis *AtWRI1* (*At3g54320*).

After cloning two amplicons of varying lengths into a pGEM T-Easy Vector (Promega and Madison, Wisconsin, United States), their respective nucleotide sequences were revealed by sequencing (Sangon Biotechnology Shanghai, China). The cDNA sequences are listed in GenBank as KU285604 and KU285605, respectively. The short form (1,479 bp) was designated as *LuWRI1a* whereas the long form (1482 bp) was known as *LuWRI1b*, with the appropriate primer sets listed in **Table 1**.

**Table 1:** Primers utilized in analyses.

Gene	Forward primer sequence	Reverse primer sequence
GAPDH LuWRI1a LuWRI1b RT-LuWRI1a RT-LuWRI1b RT-LuAct AtActin RT-AtWRI1	5′-AGGTTCTTCCCGCTCTCAAT-3′ 5′-CCACATGAAATCGCCGCCGTCAAACGAC-3′ 5′-CCACATGAAATCGCCGCCGTCAAACCAG-3′ 5′-GATGATCAAGAAGCAGCTG-3′ 5′-CGTCCAAGTCGACCAATTCC-3′ 5′-TCCAGGCCGTTCTTTCTCTA-3′ 5′-GTCTTGTTCCAGCCCTCGTTT-3′ 5′-AGACATAGATGGACTGGGAGA	5′-CCTCCTTGATAGCAGCCTTG-3′ 5′-CAAGAAACCGATGTTGTTGTTGA-3′ 5′-TTAGTTACAAGAAACCGATGTTG-3′ 5′-CATTGCCACCTCAGCGGCC-3′ 5′-GACGAGGCAGAGGATGGTTC-3′ 5′-CTGTAAGGTCACGACCAGCA-3′ 5′-GGACCTGCCTCATCATACTCG-3′ 5′-ATCGTACGTATGTGCTGCTG-3′

### Sequence analyses

The acquired WRI1 amino acid sequences were examined utilizing the bioinformatics resources available at www.ncbi.nlm.nih.gov. Clustal Omega was utilized to align the WRI1 genes of *Arabidopsis thaliana*, *Brassica napus*, *Camelina sativa*, *Gossypium hirsutum*, *Ricinus communis*, *Sesamum indicum*, *Glycine max*, *Helianthus annuus*, and *Linum usitatissimum* (www.ebi.ac.uk/Tools/msa/clustalo/). Phylogenetic tree was constructed with MEGA 6.0 using CLUSTAL W with the maximum likelihood technique and a bootstrap value of 1,000 repeats.

### Vector construction and plant transformation

*LuWRI1a* has been shown to be predominantly expressed in flax developing seeds, which were amplified using specific primers. The plasmids *pEG101-LuWRI1a* and *pBBG-LuWRI1a* were constructed. After cloning into pENTR/D-TOPO (Invitrogen), it was shuttled into the overexpression vector *pEarleyGate101* (*pEG101*) and the interference vector *pBIB-BASTA-35S-GWRNAi* (*pBBG*), with Gateway LR Clonase™ II enzyme mix (Invitrogen). All constructs were transformed into *Agrobacterium tumefaciens* GV3101, and then *Arabidopsis* were transformed using the floral dip method (Clough and Bent, 1998). Hypocotyl of flax seedling transformation was performed following a protocol described by Bretagne et al. (1994). Transformants were selected with 10 μg·ml^-1^ Basta (Sigma-Aldrich) and further confirmed by RT-PCR analysis. Gene insertion was confirmed by PCR using a 35S promoter forward primer and a gene-specific reverse primer. After sowing the T1 transgenic *Arabidopsis* seeds, Basta-resistant transgenic plants were selected by spraying with BASTA solution (10 μg·ml^-1^). The homozygous transgenic *Arabidopsis* seeds from the T3 generation were used for the subsequent experiments. The 13 *pEarlyGate101::LuWRI1a* transgenic flax plants and seven *pBIB-BASTA-35S-GWRNAi::LuWRI1a* transgenic flax plants of the T0 generations were detected by PCR and the sequencing of PCR products. The positive transgenic plants of the T1–T3 generations were confirmed by PCR; three homozygous transgenic lines *pEG101-LuWRI1a*(*LuWRI1a-OX-X*) plants and two homozygous transgenic lines *pBBG-LuWRI1a*(*LuWRI1a-iRNA-X*) plants of the T3 generations were detected.

### Quantitative Real-Time PCR Analysis

The 2SYBR Select Mixture was used for quantitative real-time polymerase chain reactions on an Eco Real-Time PCR system (Applied Biomiga). Each 50-μl reaction mixture contained 2 μl cDNA template, 25 μl of SYBR Mixture, 1 μl of each primer (10 μM), and 21 μl double-distilled water. The temperatures were as follows: 50°C for 2 min; 95°C for 10 min; and 40 cycles of 95°C for 15 s and 60°C for 1 min. Dissociation curves were generated for each reaction to ensure specific amplification. Cycle threshold (CT) values were generated from applied Illumina Eco Real-time PCR software (Illumina). The relative expression level was determined using the ^2-ΔΔ^Ct method (Rajwade et al., 2010) with the GAPDH gene as a reference. To validate the specificity of the amplification, a melting curve was constructed for each primer pair. Data were analyzed using Eco software (Illumina). Two technical replicates of each were performed.

### Seed analysis and scanning electronic microscopy

*Arabidopsis* seeds of Col-2, mutant *wri1-1*, and complemented mutant *OX : LuWRI1a/wri1-1* were photographed under light and scanning electron microscopes. The seed size and 100 *Arabidopsis* seed and 1,000 flax seed weight were measured.

### Analysis of flax seed lipid content and fatty acid composition

The method of Soxhlet extraction was used to extract total lipids. The Soxtec 8000 system was used to determine the oil content (Foss). As detailed by Rajwade et al. (Rajwade et al., 2010), fatty acid methyl esters (FAMEs) were synthesized for the study of fatty acid composition. The resultant FAMEs were filtered using an AT.KD-FFAP column with 30 m in length and 0.32 mm in diameter and had a coating thickness of 0.33 μm and then analyzed using an Agilent 7820A gas chromatograph. The GC was set to 190°C for 5 min, in which temperature was then gradually increased by 20°C min^-1^ until it reached 210°C, and held for 9 minutes. As the carrier gas, nitrogen was utilized at a flow rate of 1 ml min^-1^. The temperature of the injector port was kept at 250°C, while the FID detector temperature was kept at 300°C. The identification of fatty acid peaks was obtained by contrasting the peaks in question with the profiles of standards that were purchased from Sigma-Aldrich (USA). FA compositions were represented as the relative percentage of palmitic acid, stearic acid, oleic acid, linoleic acid, and linolenic acid. Relative fatty acid compositions were computed as a percentage of total measured fatty acids.

### Statistical analysis

Data were analyzed using the SPSS package (SPSS 20.0 Software) by one-way ANOVA. Statistical comparisons were determined using Student′s t-test to identify the differences between the transgenic lines and WT in oil content, seed size, seed weight, fatty acid composition, and relative gene expression. Significant difference test was done at a significance level of 0.05.

## Results

### Sequence and phylogenetic analyses of flax WRI1

**Figure 1:**
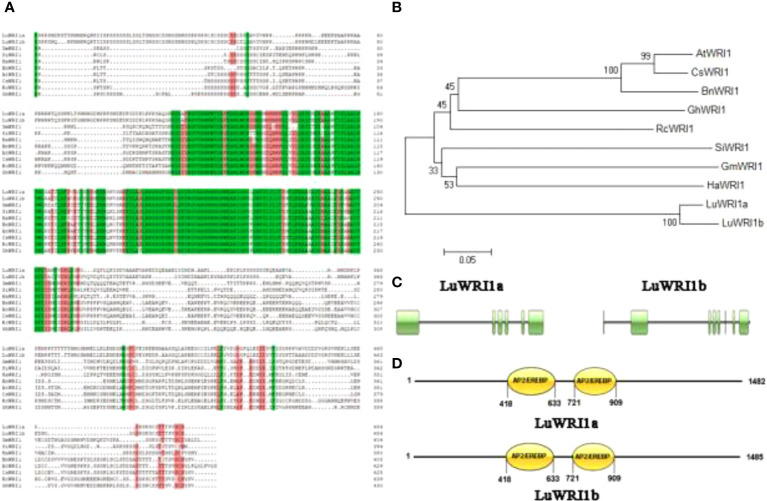
Amino acid sequence alignment and phylogenetic analysis of LuWRI1a and LuWRI1b **(A)** Sequence alignments of LuWRI1a, LuWRI1b, GmWRI1, SiWRI1, BnWRI1, HaWRI1, AtWRI1, CsWRI1, RcWRI1, GhWRI1, CeWRI1, EgWRI1, CnWRI1, and ZmWRI1 proteins. Conserved AP2/EREBP DNA binding motifs (in green) and the “VYL” motif (in orange) that activates WRI1 transcription. **(B)** Phylogenetic tree of WRI proteins. The neighbor-joining approach was used to construct a phylogenetic tree in MEGA6. AtWRI1 (AT3G54320.1), CsWRI1 (AQP31129.1), BnWRI1 (ADO16346.1), GhWRI1 (AFV61655.1), RcWRI1 (NP_001310691.1), SiWRI1 (XP_011078716.1), GmWRI1 (ADM34977.1), and HaWRI1 (AFQ93679.1). **(C)** The amino acid and nucleotide sequences of LuWRI1a and LuWRI1b were figured out. **(D)** The conserved domains of LuWRI1a and LuWRI1b. Numbers indicate the amino acid positions along the protein. Double AP2/EREBP domains were predicted in two proteins.

Two WRI1 transcripts were identified from developing flax seeds by cloning their full-length cDNA, which exhibited 89% nucleotide sequence identity. They were designated as *LuWRI1a* (KU285604) and *LuWRI1b* (KU285605). *LuWRI1a* included a 1,479-bp nucleotide and encoded 493aa with an expected molecular mass of 54.04 kDa and a pI of 5.10 (**Figure S1**, **Figure S3**). *LuWRI1b* included a 1,482-bp nucleotide and encoded 494aa with an expected molecular mass of 53.95 kDa and a pI of 5.23 (**Figure S2**, **Figure S4**). The two scaffolds had 76.5% homology to *AtWRI1* in amino acid sequences. Both of the proteins had two typical AP2/ERF DNA-binding domains at 418–633 and 721–909 amino acids (**Figures 1A, C, D**) and showed a high sequence conservative with the APETALA2/EREBP domain of other members of the AP2 family. In order to elucidate the evolutionary correlation between the two LuWRI1 isoforms and WRI1 homologue proteins from other species, phylogenetic analysis was carried out, and the result indicates that *LuWRI1a* and *LuWRI1b* fell into the same clade. However, the WRI1s that have been currently identified fell into another clade (**Figure 1B**).

### *LuWRI1a* is predominantly expressed in developing seeds of flax

**Figure 2:**
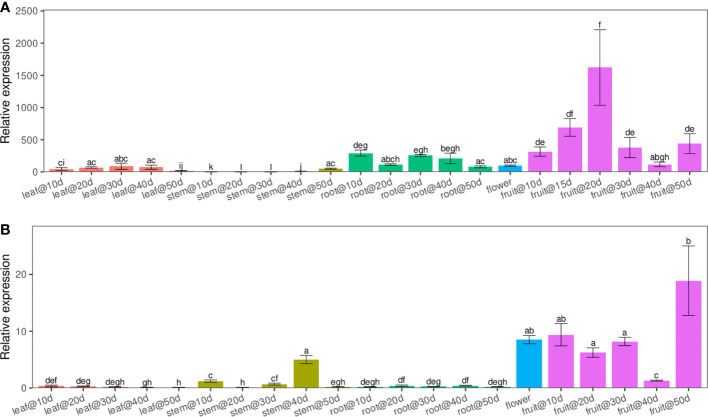
*LuWRI1a* and *LuWRI1b* profile expressions of flax plant. By qRT-PCR, we were able to determine the expressions profiling of *LuWRI1a* and *LuWRI1b* in the primary flax organs. R1–R5, root in the 10th to 50th days after flowering; S1–S5, stem in the 10th to 50th days after flowering; L1–L5, leaves in the 10th to 50th days after flowering; F, the first three opened flowers; 10D–50D, developing seed stages. Error bars represent SD (n = 3). Student′s t-test was utilized. These identical letters revealed that there is not a statistical significance (P< 0.05).

Using quantitative real-time PCR (qRT-PCR) with transcript-specific primers, we analyzed the expression patterns of *LuWRI1a* and *LuWRI1b* in several flax tissues, including roots, stems, leaf, and seeds, across a variety of developmental phases. The results revealed that *LuWRI1a* and *LuWRI1b* expressed ubiquitously in all tissues tested, while higher transcript levels of *LuWRI1a* and *LuWRI1b* in developing seeds were observed relative to the other tissues (**Figure 2**). Furthermore, the *LuWRI1a* expression level was significantly higher than that of *LuWRI1b* at the developmental period of the seeds (**Figure 2**). After 20 DAF, there was a significant rise in *LuWRI1a* expression levels (**Figure 2A**), whereas *LuWRI1b* were observed after 50 DAF (**Figure 2B**).

### Functional analysis of *LuWRI1a* in *A. thaliana* seeds

**Figure 3:**
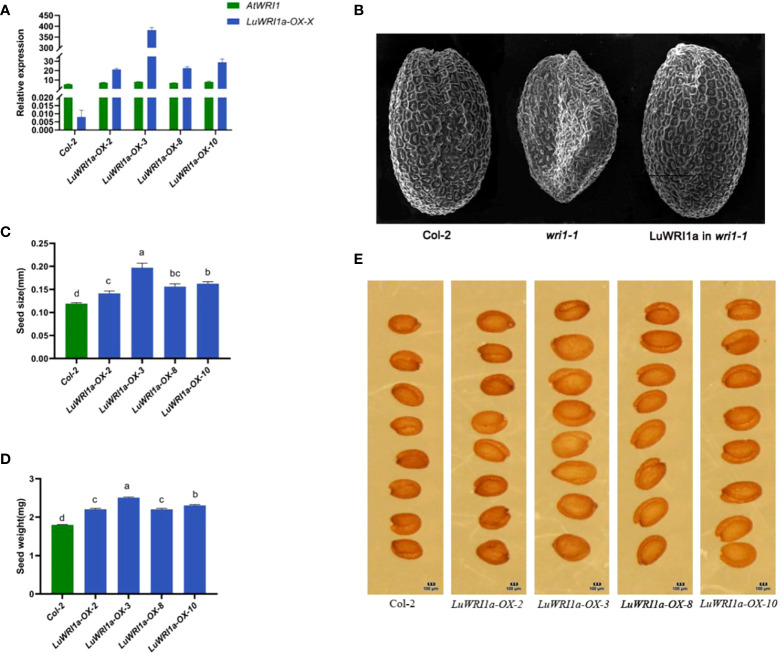
Functional analysis of *LuWRI1a* in *A. thaliana* seeds. **(A)** Expression profiles of *LuWRI1a* were established by qRT-PCR in the seeds of *Arabidopsis* transgenic lines and null transgenic (Col-2). *LuWRI1a* overexpression transgenic *Arabidopsis* lines were *LuWRI1a-OX-X*, including *LuWRI1a-OX-2*, *LuWRI1a-OX-3*, *LuWRI1a-OX-8*, and *LuWRI1a-OX-10*. **(B)** The wrinkled seed phenotype of *Arabidopsis* was enhanced by the *LuWRI1a* gene. Images of mature seeds of the null transgenic (Col-2), mutant (*wri1-1*), and *wri1-1* expressing *LuWRI1a*. Scanning electron microscopy was utilized in order to investigate the seed phenotype. Bar = 200 μm. **(C)** Size analysis of null transgenic and transgenic *Arabidopsis* seeds. Error bars represent SD (n = 8). **(D)** Seed weight analysis of null transgenic and *LuWRI1a* transgenic lines, mg/100 per. Error bars represent SD (n = 9). **(E)** Images of wild-type and transgenic *LuWRI1a* in null transgenic background. Bar = 100 μm.

To elucidate the role of *LuWRI1a*, *pEG101-LuWRI1a* was overexpressed in *Arabidopsis* wild-type plants (Col-2). Primers designed from inside the *LuWRI1a* gene were used for the quantitative RT-PCR analysis. To measure *LuWRI1a* transcript levels in seeds, we chose four plants that tested positive for PCR from overexpression transgenic *Arabidopsis* lines. As shown in **Figure 3A**, four lines (*LuWRI1a-OX-X*) showed a high expression level of *LuWRI1a*.

Furthermore, a complementary experiment was carried out against the *Arabidopsis wri1-1* mutant background. Homozygous *Arabidopsis wri1-1* mutants (Baud et al., 2007) were transformed with flax *LuWRI1a* cDNA, under the constitutive cauliflower mosaic virus (CaMV) 35S promoter. As depicted in **Figure 3B**, microscopy analysis of fully grown, dried seeds expressing *LuWRI1a* revealed a restoration of the wrinkled seed phenotype seen in *wri1-1* mutant seeds.

Seed size and weight were compared between non-transgenic and transgenic lines to investigate if there was a difference due to the expression of *LuWRI1a* in transgenic *Arabidopsis*. The seed size from the four transgenic *Arabidopsis* lines increased by 18.9% to 65.8% compared with that of wild-type lines (**Figures 3C, E**). Furthermore, the seed weight of line *LuWRI1a-OX-3* increased by 39.4% relative to that of WT lines (**Figure 3D**). Seed size and weight were shown to be considerably greater in transgenic lines compared to WT utilizing statistical analysis.

### Overexpression of *LuWRI1a* in *Arabidopsis*-enhanced seed oil contents

**Figure 4:**
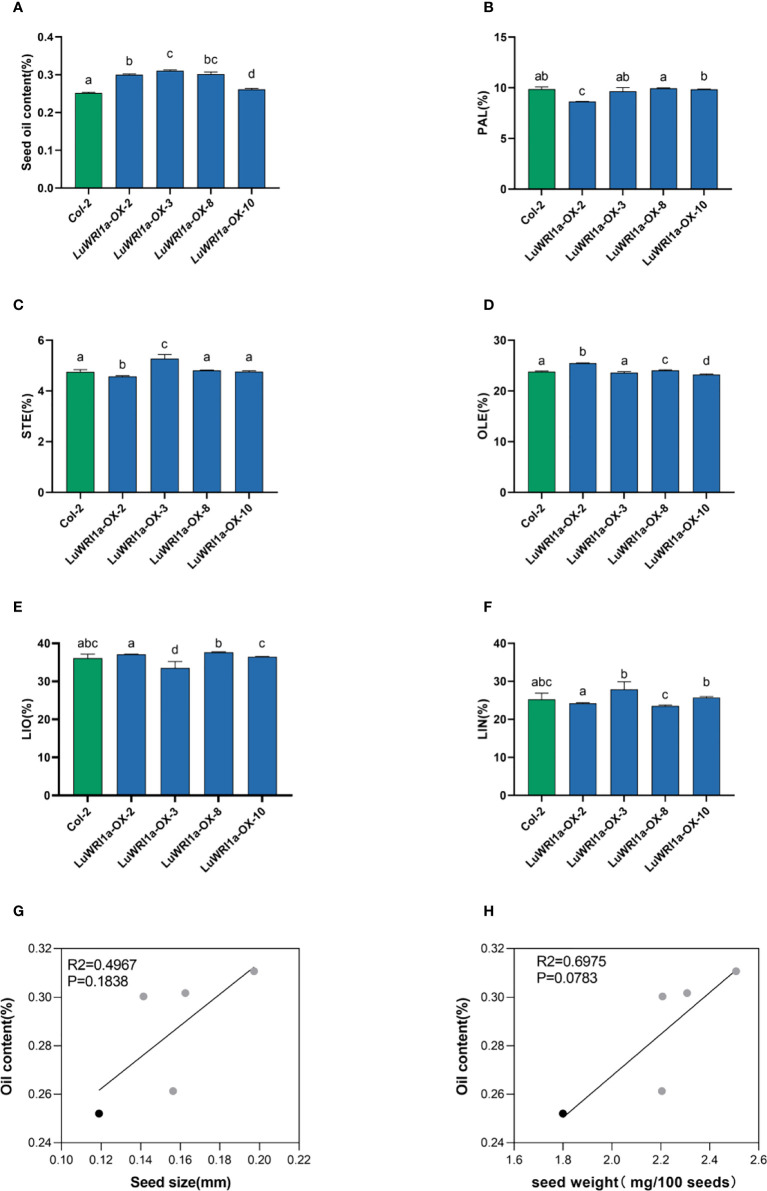
Overexpression of *LuWRI1a* in *Arabidopsis* seedlings enhanced oil content. **(A)** Transgenic *LuWRI1a* overexpression of *Arabidopsis*. Seed oil contents of four T3 homozygous *LuWRI1a*-overexpressing transgenic plants (*LuWRI1a-OX-X*) and null transgenic (Col-2). Error bars represent SD (n = 3). **(B-F)**. Summary of the five major fatty acid profiles in the seeds of null transgenic (Col-2) and four T3 homozygous *LuWRI1a*-overexpressing transgenic plants (*LuWRI1a-OX-X*). Palmitic acid (PAL), stearic acid (STE), oleic acid (OLE), linoleic acid (LIO), and linolenic acid (LIN) are all fatty acids. Error bars represent SD (n = 4). **(G)** Correlative analysis of the oil content and seed size(mm) in null transgenic (black) and transgenic lines of flax (gray). **(H)** Correlative analysis of the oil content and seed weight(mg/100 seeds) in null transgenic (black) and transgenic lines of flax (gray).

Oil contents in transgenic seeds of *Arabidopsis* WT plants at the background were measured. The results showed that overexpression of *LuWRI1a* greatly increase oil contents. In detail, the oil contents of seed (dry weight, DW) in the four transgenic seeds were 30.0%, 31.1%, 26.1%, and 30.2% (**Figure 4A**), which showed a significant increase by 19.2%, 23.3%, 3.7%, and 19.7% compared with their corresponding null segregants. The statistical test indicated a significant difference in seed oil contents (P< 0.01) between three transgenic lines and WT plants (**Figure 4A**).

The impact of *LuWRI1a* expression on FA composition in *Arabidopsis* seeds was further investigated. The results showed no statistical variation in fatty acid composition among transgenic lines seeds and WT plants (**Figures 4B–F**). Furthermore, correlation analysis showed no significant relation between seed oil content and seed size or seed weight of the transgenic lines (**Figures 4G, H**).

### *LuWRI1a* affected the morphological characteristics and oil accumulation of flax seeds

**Figure 5:**
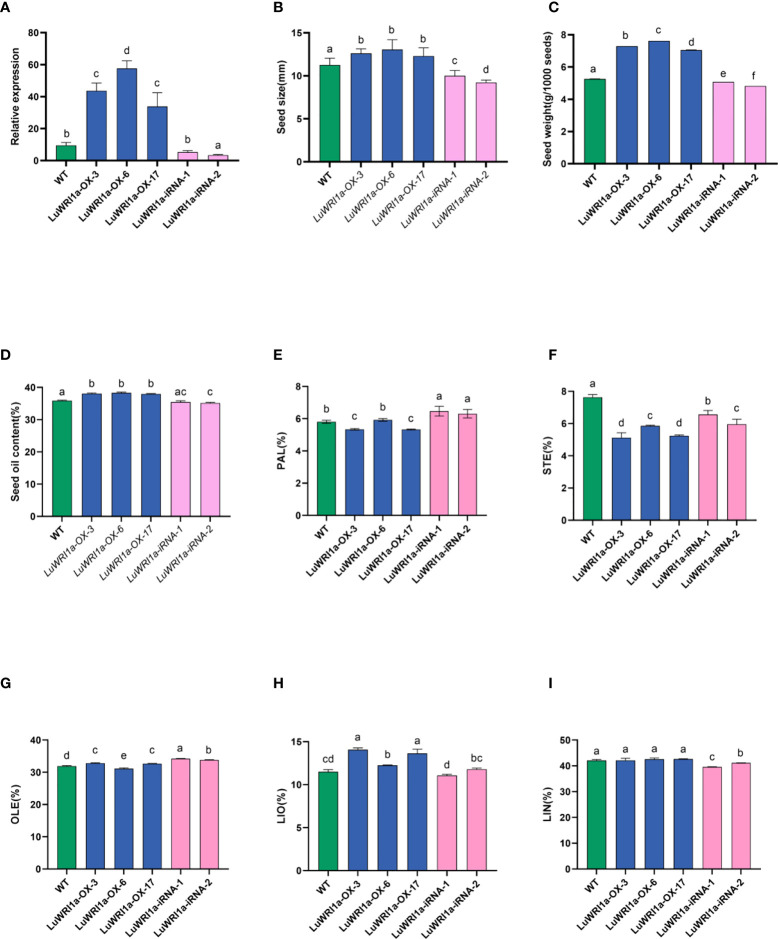
Effects of overexpression of *LuWRI1a* on seed weight, seed size, and seed oil content of flax. **(A)** *LuWRI1a* expression transcript was assayed in mature seeds from transgenic flax, normalized by that of actin. **(B)** Comparison of the sizes of non-transgenic and genetically modified flax seeds. Error bars represent SE (n = 10). **(C)** 1,000-seed weight of untransformed flax seeds and five transgenic lines. Error bars represent SE (n = 3). **(D)** Oil contents of untransformed flax seeds (WT), three independent T3 *pEarlyGate101::LuWRI1a* transgenic lines (*LuWRI1a-OX-X*), and two independent T3 *pBIB-BASTA-35S-GWRNAi::LuWRI1a*(*LuWRI1a-iRNA-X*). The oil contents were determined by the Soxhlet method. DW, dry weight. Error bars represent SE (n = 3). **(E–I)** Fatty acid composition from seeds of (WT) and transgenic plants were examined by GC assays. Error bars denote SE (n = 3).

Using the mRNA that was extracted from flax seeds at 20DAF as a template, flax full-length *LuWRI1a* cDNA was synthesized by reverse transcription PCR and then ligated on overexpression vector pEG101 and interference vector pBBG. Five independent homozygous transgenic flax of the T3 generations, i.e., three *LuWRI1a-OX-X* lines and two *LuWRI1a-iRNA-X* lines, were from different T0 generations and used for further analysis. As shown in **Figure 5A**, three *LuWRI1a-OX-X* lines showed a higher expression level of *LuWRI1a* while two *LuWRI1a-iRNA-X* lines showed a lower expression level compared to the WT plants (null transgenic).

To examine whether *LuWRI1a* overexpression affects the morphological characteristics of flax seeds, the seed size and 1,000-seed weight of null transgenic and transgenic lines were evaluated. In comparison to WT, the seed size of three *LuWRI1a-OX-X* lines has increased by approximately 9.2% to 16.0% (**Figure 5B**, **S5**). However, the seed size of two *LuWRI1a-iRNA-X* lines was decreased by 11.1% and 18.2% compared with the WT plants, respectively (**Figure 5B**). The average seed size of the WT plants was approximately 11.3 mm^2^, whereas they were 12.6, 13.1, and 12.3 mm^2^ in the three transgenic lines (**Figure 5B**). The 1,000-seed weight of the WT plants was approximately 5.3 g, whereas they were 7.3, 7.6, and 7.1 g in three *LuWRI1a-OX-X* lines (**Figure 5C**). In comparison with the WT plants, the 1,000-seed weight of three *LuWRI1a-OX-X* transgenic lines was significantly increased by 34.1%, 38.5%, and 44.8%, whereas that of two *LuWRI1a-iRNA-X* lines was significantly decreased by 3.6% and 8.4% (**Figure 5C**).

The seed oil contents of *LuWRI1a-OX-X*, *LuWRI1a-iRNA-X*, and the WT plants were further measured. The results showed that the seed oil contents of three *LuWRI1a-OX-X* lines (*LuWRI1a-OX-3*, *LuWRI1a-OX-6*, and *LuWRI1a-OX-17*) were significantly increased by 6.1%, 6.8%, and 5.8% compared with the WT plants (**Figure 5D**). However, the seed oil contents of two *LuWRI1a-iRNA-X* lines (*LuWRI1a-iRNA-1* and *LuWRI1a-iRNA-2*) were decreased by 1.3% and 2.1% relative to the WT plants (**Figure 5D**).

Our findings also showed that overexpression of *LuWRI1a* in flax did not affect polyunsaturated fatty acids of TAG, especially the linolenic acid content. As shown in **Figures 5E–I**, the level of stearic acid (STE) was decreased and the linoleic acid (LIO) level was increased in *LuWRI1a-OX-X* seeds compared with the WT plants.

### The association of the oil content, phenotype of seeds, and expression level of *LuWRI1a*

**Figure 6:**
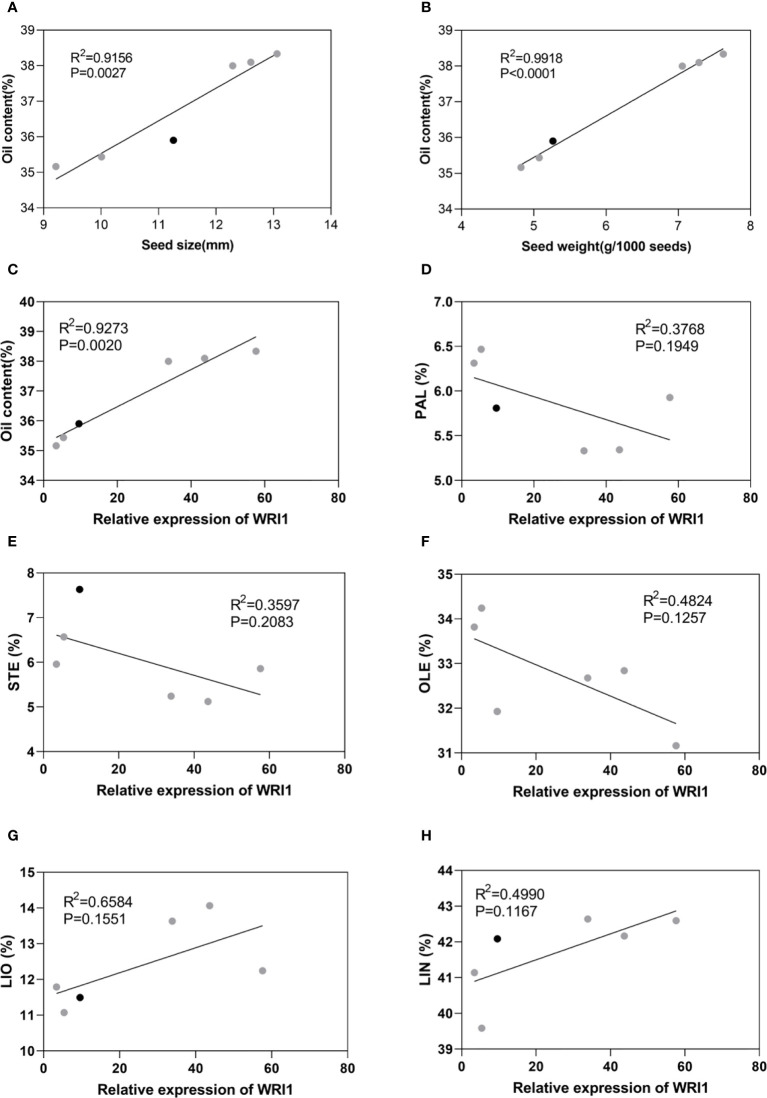
Correlation analysis in WT (black) and transgenic lines of flax (gray). **(A)** Correlation analysis between seed oil content and seed size. **(B)** Correlation analysis between seed oil content and seed weight. **(C)** Correlation analysis between seed oil content and expression levels of WRI1. **(D–H)** Correlation analysis between relative expression of WRI1 and PAL(%), STE(%), OLE(%), LIO(%), and LIN(%).

Correlation analyses indicated that *LuWRI1a-OX-X* line seed oil content was positively correlated with the seed size and weight (**Figures 6A, B**). In particular, seed weight was positively associated with oil content, suggesting that the oil accumulation of transgenic flax seeds is mainly attributed to the seed weight (**Figure 6B**). Furthermore, we analyzed the correlation between *LuWRI1a* expression level and oil content or five mainly fatty acids. The expression of WRI1 was positively associated with the oil content (**Figures 6C**), but not significantly related with the content of the five mainly fatty acids (**Figures 6D–H**).

### Analyzing the transcriptions of genes involved in FA synthesis, TAG assembly, and LAFL proteins

**Figure 7:**
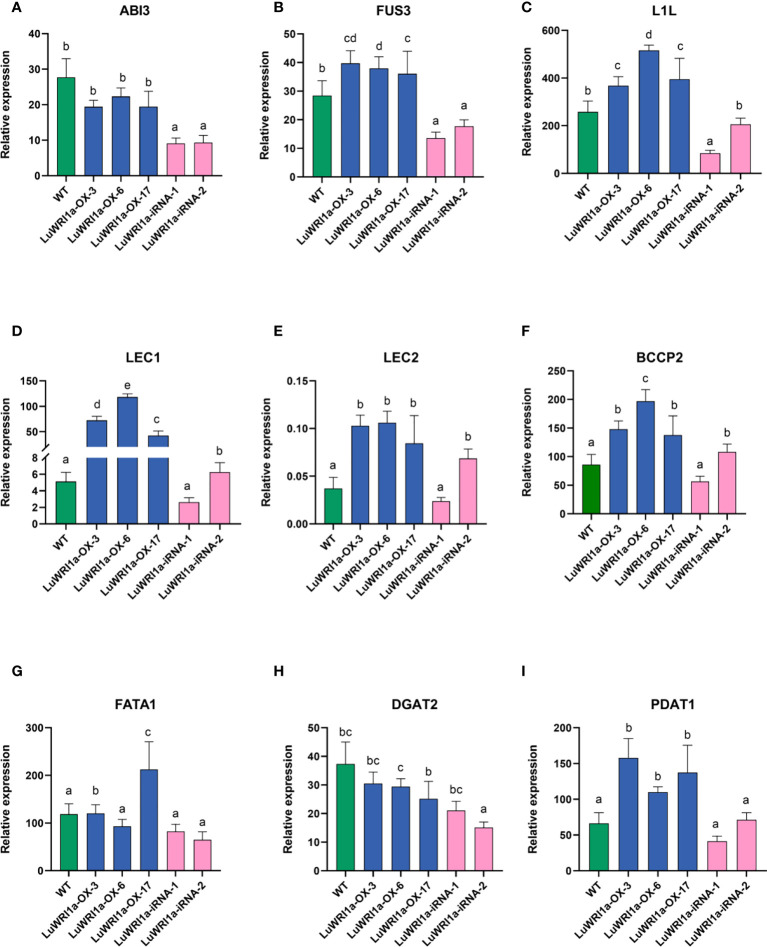
Relative gene expression levels of the ABI3 **(A)**, FUS3 **(B)**, L1L **(C)**, LEC1 **(D)**, LEC2 **(E)**, BCCP2 **(F)**, FATA1 **(G)**, DGAT2 **(H)**, and PDAT1 **(I)** in WT, *LuWRI1a-OX-X*, and *LuWRI1a-iRNA-X*. The expression of GAPDH was used as an internal reference, and the results were standardized relative to that. Error bars represent SD (n = 3).

Previous studies have reported that it is a rapid accumulation period of seed oil after 20 flowering days in flax (Li et al., 2019). We analyzed immature seeds at developmental stages (20 DAP). The qRT-PCR analyzed include the key genes in the FA biosynthesis and TAG accumulation (**Figure 7**), such as those encoding BCCP subunit and thioesterase (BCCP2 and FATA1), diacylglycerol acyltransferase, and phosphatidylcholine: diacylglycerol acyltransferase (DGAT2 and PDAT1), and five TFs are referred to as LAFL proteins (ABI3, FUS3, L1L, LEC1, and LEC2) (**Table S1**), which is absolutely necessary for the formation of store lipids as the seed matures. *LuWRI1a* overexpression in transgenic line seedlings increased the expression of the aforementioned genes, except for ABI3 and DGAT2. Notably, expressions of L1L, BCCP2, and LEC1 were significantly upregulated than in the WT. Meanwhile, the levels of these genes′ transcripts among seeds of *LuWRI1a-iRNA-X* lines were lower than WT mostly.

### The relationship regarding the oil content and related gene expression level

In the transgenic strains, the expression of WRI1 was associated with the expression of genes in FA biosynthesis, TAG assembly, and LAFL proteins, as shown in **Table 2**. The WRI1 expression level revealed a highly significantly strong association between the expression levels of L1L, LEC1, and BCCP2 (P ≤ 0.01) and revealed a significantly positive association between FUS3 and LEC1 (P ≤ 0.05).

**Table 2:** Pearson’s correlation analysis between expression levels of WRI1 and expression levels of related genes.

	WRI1	ABI3		FUS3	L1L	LEC1	LEC2	BCCP2	FATA1	DGAT2
ABI3	0.469									
FUS3	0.884*	0.723								
L1L	0.923**	0.618		0.915*						
LEC1	0.975**	0.391		0.790	0.894*					
LEC2	0.869*	0.262		0.810	0.877*	0.858*				
BCCP2	0.924**	0.403		0.819*	0.957**	0.941**	0.947**			
FATA1	0.324	0.425		0.561	0.412	0.121	0.255	0.210		
DGAT2	0.389	0.937**		0.620	0.430	0.310	0.099	0.220	0.303	
PDAT1	0.787				0.765	0.672	0.868*	0.740	0.590	0.278

“*” denotes a significant association (P ≤ 0.05), whereas “**” denotes an extremely significant correlation (P ≤ 0.01).

## Discussion

Flax seed is an oil seed crop that contains the richest plant source of linolenic acid (LIN), which has potential health benefits such as in reduction of cardiovascular disease, cholesterol, triglyceride, cancer, and autoimmunity (Gebauer et al., 2006, Goyal et al., 2016). There has been a lot of focus on improving the oil content and quality of flax seeds in breeding improvement. Throughout several higher plants, oil accumulation in seeds is tightly regulated by complicated regulatory networks that coordinate a wide range of environmental and developmental signals. One of the most important factors affecting FA availability for TAG production is transcriptional regulation. However, till now the processes underpinning the regulation of TFs and regulatory networks on the overall quantity of oil store in plant seeds remain unclear. In order to understand how flax seed oil accumulates at the molecular level, it is crucial to identify the fundamental regulators of seed oil accumulation. WRI1 is an AP2 transcription factor that regulates the key expression genes in the glycolytic and fatty acid biosynthetic pathways, hence playing a crucial role in the transcriptional regulation of TAG synthesis (Kong and Ma, 2018b). In this study, we found two *LuWRI1* cDNAs in flax, both of which were of varying lengths, named *LuWRI1a* and *LuWRI1b*. *LuWRI1a* had 77% homology and *LuWRI1b* had 76% homology with *Arabidopsis AtWRI1* of amino acid sequencings. Two “VYL” residues (typical of AP2/EREBP DNA-BDs) were identified in both proteins. WRI1 is an AP2-type transcription factor in *Arabidopsis*, and mutations in either the “Y” or “L” residues completely impaired transcription activation in the mutated population. *LuWRI1a* encodes functional proteins; it can complement the wrinkled phenotype. *LuWRI1a* and *LuWRI1b* preferentially expressed in developing seeds compared to other tissues; the expression of *LuWRI1a* was much higher than that of *LuWRI1b* at the developmental period of the seeds. The 20 days after flowering (20D) is the period of rapid accumulation of seed oil, and the 50 days after flowering (50D) is the period of seed maturity (Li et al., 2019). *LuWRI1a* reached its highest expression at 20D, and *LuWRI1b* reached its highest expression at 50D. The two flax genes *LuWRI1a* and *LuWRI1b* might not play the same role in flax plants; *LuWRI1b* may be a complement to *LuWRI1a* in the function of seed oil accumulation during the period of seed maturity. Therefore, we selected *LuWRI1a* which is very highly expressed during the seed development period for further study.

Overexpression of WRI1 has been demonstrated to increase oil content in seeds in previous research. For example, an about (10–20)% rise in seed oil content was detected in *AtWRI1*-overexpressing transgenic *Arabidopsis* (Cernac and Benning, 2004). The seed oil concentration was enhanced by approximately 31% when *ZmWRI1* was overexpressed in maize (Shen et al., 2010) and was increased by approximately 20% and 10% when *BnWRI1* was overexpressed in *Arabidopsis* and oilseed rape, respectively (Liu et al., 2010). However, the role of WRI1 in regulating TAG synthesis and oil accumulation in flax seeds remains unclear. In our study, we demonstrated that *LuWRI1a* also enhanced the storage oil contents. When overexpressing *LuWRI1a* in *Arabidopsis* and flax, oil content was increased by approximately 23.3% and 6.8%, respectively. However, the seed oil contents of *LuWRI1a-iRNA-2* was decreased by 2.1% compared with the WT plants.

High yield and quality have always been the main objectives of applied and basic crop research; the conflict is a universal scientific problem in different crops. Previous research suggested that the oil content might be increased by the overexpression of certain quality-related genes, either through constitutive expression or through increased expression in dicots and monocots. However, this led to a range of diseases of agronomic traits (Shen et al., 2010). For example, seed weight was lowered by 29% at maturity due to the increased oil content in the endosperm by fivefold on a per grain, which was achieved by the expression of *AsWRI1* (Grimberg et al., 2020). *OsNPR1* overexpression may improve resistance in rice, but at the expense of normal rice development (Xu et al., 2017). It is crucial for agricultural production to identify positive regulators of fatty acid biosynthesis. In the study, *LuWRI1a* overexpression in transgenic *Arabidopsis* and flax resulted in larger seeds with a higher weight as well as increased storage oil levels. Correlation analyses indicated that content of seed oil was highly significantly associated with the 1,000-seed weight; it is a decisive factor in the yield of agronomic traits. However, a previous study revealed a convoluted correlation between oil rise and seed weight. The seed-specific expression of *TmDGAT1* caused an increase in seed weight in *Arabidopsis*, but not in *B. napus* (Xu et al., 2008). Overexpressing *AtHb2* in *Arabidopsis* lines did not affect seed weight (Vigeolas et al., 2011). More research in future is needed to determine if and how WRI1 expression levels and patterns relate to seed weight and oil content. We examined the main fatty acid composition in seed oil to investigate if *LuWRI1a* overexpression had any effect on oil quality. The results showed no considerable changes in linolenic acid levels. Furthermore, there was a positive and statistically significant correlation between WRI1 expression and oil concentration in transgenic flax seeds, but no such correlation existed for the five primary fatty acids. The results proved that increasing *LuWRI1a* expression in flax increased seed oil content and the 1,000-seed weight of seeds, while not altering the oil quality.

According to previous research, WRI1 has been observed to act as the major regulator of fatty acid biosynthesis in which it promotes direct SUS2, BCCP1, GLB1, ROD1, and KASI expression during *Arabidopsis* seed development (Ruuska et al., 2002; Baud et al., 2009; Maeo et al., 2009; To et al., 2012). We investigated the expression levels of TFs that influence the expression of genes involved in FA synthesis and TAG assembly, as well as LAFL genes in transgenic flax. There are highly complex regulatory networks formed by LAFL genes that involved seed maturation and storage material accumulation. Several genes, such as LEC1 and LEC2, are positive upstream regulators of WRI1 ABI3, and FUS3 and, together with ABI3, FUS3 and WRI1, are responsible for lipid accumulation and protein storage in seeds (Mu et al., 2008). We did observe that BCCP2, FATA1, FUS3, L1L, LEC1, LEC2, and PDAT1 would upregulate in flax transgenic plants overexpressing LuWRI1a. We found that L1L, BCCP2, and LEC1 displayed high expression levels in developing flax seeds, and the expression level of *LuWRI1a* was highly significantly positively associated between L1L, LEC1, and BCCP2 (P ≤ 0.01). Our studies suggest that L1L possibly acts as a new interacting partner of WRI1, increasing the transcription of genes involved in fatty acid production that are targeted by WRI1, together with WRI1. The interesting questions need to be investigated further. Previous studies have shown that the WRI1-regulated FA biosynthesis pathway is independent of MYB96 regulation of TAG accumulation, while WRI1 mainly regulates glycolysis and the late FA biosynthesis; MYB96 stimulates the TAG assembly process (Lee et al., 2018). The present study also revealed that there was no significantly positive association between the expression levels of DGAT2 and PDAT1; our evidence also indicated that WRI1-regulated FA biosynthesis is independent of TAG accumulation. This study provides evidence that LuWRI1a is a key transcription factor in regulating the expression of genes throughout the FA biosynthesis pathway.

In decades past, flax varieties of high content seed oil have played a significant role in vegetable oil production. Our study suggests that the function of *LuWRI1a* in the transcriptional regulation of TAG accumulation is conserved. As such, this study showed that *LuWRI1a* acts as a possible role in oil bioengineering for flax in the future. Moreover, there were no deleterious effects on oil quality and yield.

## Conclusion

We characterized the WRI1 family gene *LuWRI1a* and *LuWRI1b* in flax (*Linum usitatissimum* L.). Overexpression of *LuWRI1a* in *Arabidopsis* and flax enhanced the weight and size of seeds besides oil contents significantly. Correlation analyses indicated that the seed oil content was highly significantly related to 1,000-seed weight, while not altering seed oil quality. Meanwhile, we investigated some transcription factor (TF) expression levels that regulate the expression of genes involving TAG assembly and FA biosynthesis, as well as LAFL genes in transgenic flax. We found that the expression level of LuWRI1a has a highly statistically positive association between L1L, LEC1, and BCCP2, suggesting that *LuWRI1a* plays a role in increasing oil biosynthesis in flax seed as it upregulates target genes of FA biosynthesis. Eventually, the transgenic expression of *LuWRI1a* in flax enhances seed oil content with no discernible impact on seed oil quality or seed production compared to controls. Thus, it seems that seeds with a moderately high oil content do not experience a loss in seed and oil output. This opens the door for the characterization of the *LuWRI1a* gene on the positive regulation of oil content in flax.

## Data availability statement

The datasets presented in this study can be found in Arabidopsis Information Resource database (www.arabidopsis.org) or GenBank/EMBL databases. The names of the accession number(s) can be found in the article.

## Author contributions

WL performed the experiments, carried out most data analysis, and wrote the manuscript. WL, LW, YQ, YX, WZ, ZD, and JZ. advised on the experiments and data analysis. J.Z. designed the entire experiment and corrected the manuscript. All authors contributed to the article and approved the submitted version.

## Funding

This work was supported by grants from the National Natural Science Foundation of China (31460388), China Agriculture Research System (CARS-14-1-05), Biological Breeding Special Project of Gansu Academy of Agricultural Sciences (2022GAAS04/2020GAAS08), Natural Science Foundation of Gansu (21JR1RA354/21JR7RA722).

## Acknowledgments

Many thanks are given to Professor Christoph Benning (East Lansing, Michigan, American) for kindly providing *Arabidopsis thaliana wri1-1* mutants.

## Conflict of interest

The authors declare that the research was conducted in the absence of any commercial or financial relationships that could be construed as a potential conflict of interest.​

## Publisher’s note

All claims expressed in this article are solely those of the authors and do not necessarily represent those of their affiliated organizations, or those of the publisher, the editors and the reviewers. Any product that may be evaluated in this article, or claim that may be made by its manufacturer, is not guaranteed or endorsed by the publisher.
