# Supplementary material for: Overexpression of WRINKLED1 improves the weight and oil content in seeds of flax (Linum usitatissimum L.)
Source: Front Plant Sci. 2022 Sep 30;13:1003758. doi: 10.3389/fpls.2022.1003758 (PMC9562325; doi:10.3389/fpls.2022.1003758)
Supplement: Supplementary file 1 [file DataSheet_1.docx]

Supplementary Material

# Supplementary Figures and Tables

#
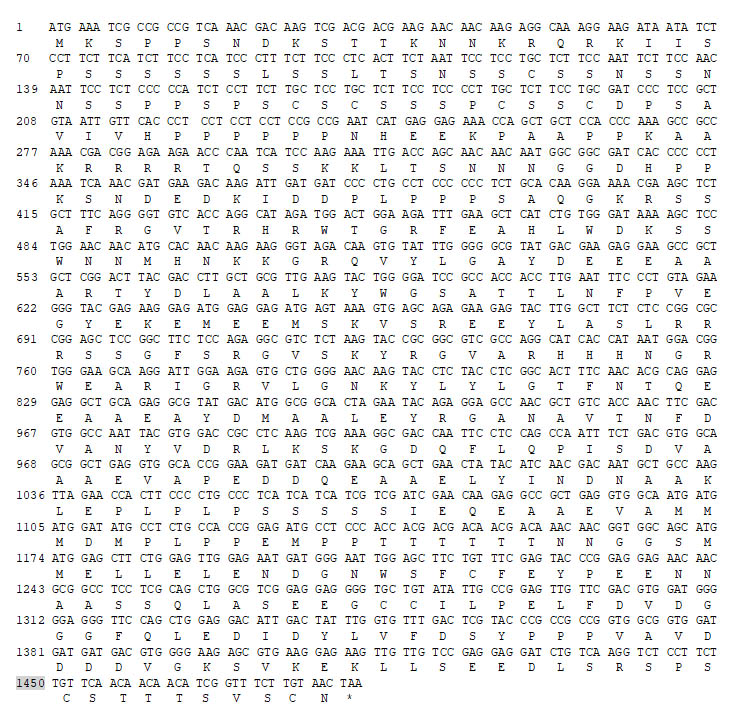


**Supplementary Figure 1**. Detailed nucleotide and deduced amino acid sequences of LuWRI1a


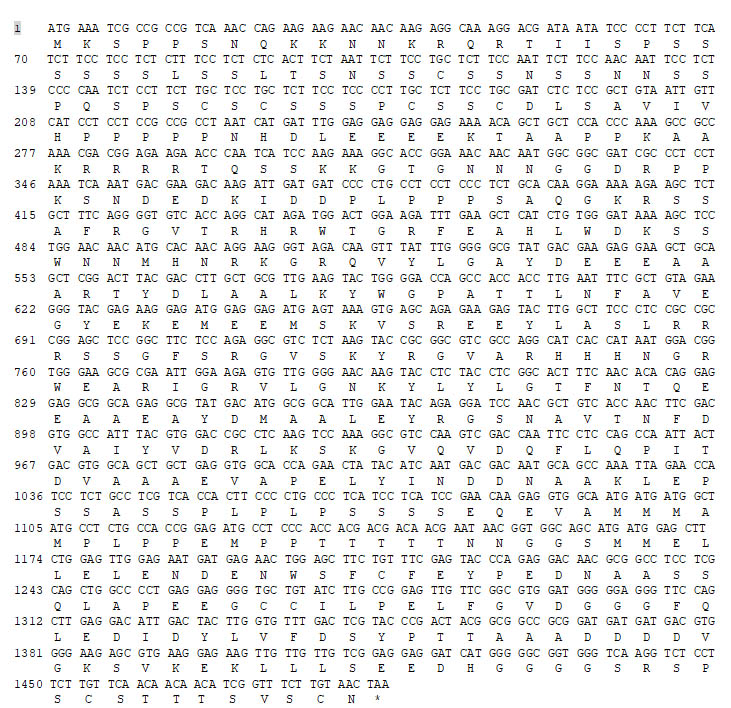


**Supplementary Figure 2.** Detailed nucleotide and deduced amino acid sequences of LuWRI1b

CGGTGGGCAAATAATGATTTTATTTTGACTGATAGTGACCTGTTCGTTGCAACAAATTGATGAGCAATGCTTTTTTATAATGCCAACTTTGTACAAAAAAGCAGGCTCCGCGGCCGCCCCCTTCACCATGAAATCGCCGCCGTCAAACGACAAGTCGACGACGAAGAACAACAAGAGGCAAAGGAAGATAATATCTCCTTCTTCATCTTCCTCATCCCTTTCTTCCCTCACTTCTAATTCCTCCTGCTCTTCCAATTCTTCCAACAATTCCTCTCCCCCATCTCCTTCTTGCTCCTGCTCTTCCTCCCCTTGCTCTTCCTGCGATCCCTCCGCTGTAATTGTTCACCCTCCTCCTCCTCCGCCGAATCATGAGGAGAAACCAGCTGCTCCACCCAAAGCCGCCAAACGACGGAGAAGAACCCAATCATCCAAGAAATTGACCAGCAACAACAATGGCGGCGATCACCCCCCTAAATCAAACGATGAAGACAAGATTGATGATCCCCTGCCTCCCCCCTCTGCACAAGGAAAACGAAGCTCTGCTTTCAGGGGTGTCACCAGGCATAGATGGACTGGAAGATTTGAAGCTCATCTGTGGGATAAAAGCTCCTGGAACAACATGCACAACAAGAAGGGTAGACAAGTGTATTTGGGGGCGTATGACGAAGAGGAAGCCGCTGCTCGGACTTACGACCTTGCTGCGTTGAAGTACTGGGGATCCGCCACCACCTTGAATTTCCCTGTAGAAGGGTACGAGAAGGAGATGGAGGAGATGAGTAAAGTGAGCAGAGAAGAGTACTTGGCTTCTCTCCGGCGCCGGAGCTCCGGCTTCTCCAGAGGCGTCTCTAAGTACCGCGGCGTCGCCAGGCATCACCATAATGGACGGTGGGAAGCAAGGATTGGAAGAGTGCTGGGGAACAAGTACCTCTACCTCGGCACTTTCAACACGCAGGAGGAGGCTGCAGAGGCGTATGACATGGCGGCACTAGAATACAGAGGAGCCAACGCTGTCACCAACTTCGACGTGGCCAATTACGTGGACCGCCTCAAGTCGAAAGGCGACCAATTCCTCCAGCCAATTTCTGACGTGGCAGCGGCTGAGGTGGCACCGGAAGATGATCAAGAAGCAGCTGAACTATACATCAACGACAATGCTGCCAAGTTAGAACCACTTCCCCTGCCCTCATCATCATCGTCGATCGAACAAGAGGCCGCTGAGGTGGCAATGATGATGGATATGCCTCTGCCACCGGAGATGCCTCCCACCACGACGACAACGACAAACAACGGTGGCAGCATGATGGAGCTTCTGGAGTTGGAGAATGATGGGAATTGGAGCTTCTGTTTCGAGTACCCGGAGGAGAACAACGCGGCCTCCTCGCAGCTGGCGTCGGAGGAGGGGTGCTGTATATTGCCGGAGTTGTTCGACGTGGATGGGGGAGGGTTCCAGCTGGAGGACATTGACTATTTGGTGTTTGACTCGTACCCGCCGCCGGTGGCGGTGGATGATGATGACGTGGGGAAGAGCGTGAAGGAGAAGTTGTTGTCCGAGGAGGATCTGTCAAGGTCTCCTTCTTGTTCAACAACAACATCGGTTTCTTGTAACAAGGGTGGGCGCGCCGACCCAGCTTTCTTGTACAAAGTTGGCATTATAAGAAAGCATTGCTTATCAATTTGTTGCAACGAACAGGTCACTATCAGTCAAAATAAAATCATTATTGCCATCCAGCTGAATCCCCCTAAAAAGGG

**Supplementary Figure 3.** Full-length cDNA sequence of *LuWRI1a*

GGGGACGAGTGGGCAAATAATGATTTTATTTTGACTGATAGTGACCTGTTCGTTGCAACAAATTGATGAGCAATGCTTTTTTATAATGCCAACTTTGTACAAAAAAGCAGGCTCCGCGGCCGCCCCCTTCACCATGAAATCGCCGCCGTCAAACCAGAAGAAGAACAACAAGAGGCAAAGGACGATAATATCCCCTTCTTCATCTTCCTCCTCTCTTTCCTCTCTCACTTCTAATTCTTCCTGCTCTTCCAATTCTTCCAACAATTCCTCTCCCCAATCTCCTTCTTGCTCCTGCTCTTCCTCCCCTTGCTCTTCCTGCGATCTCTCCGCTGTAATTGTTCATCCTCCTCCGCCGCCTAATCATGATTTGGAGGAGGAGGAGAAAACAGCTGCTCCACCCAAAGCCGCCAAACGACGGAGAAGAACCCAATCATCCAAGAAAGGCACCGGAAACAACAATGGCGGCGATCGCCCTCCTAAATCAAATGACGAAGACAAGATTGATGATCCCCTGCCTCCTCCCTCTGCACAAGGAAAAAGAAGCTCTGCTTTCAGGGGTGTCACCAGGCATAGATGGACTGGAAGATTTGAAGCTCATCTGTGGGATAAAAGCTCCTGGAACAACATGCACAACAGGAAGGGTAGACAAGTTTATTTGGGGGCGTATGACGAAGAGGAAGCTGCAGCTCGGACTTACGACCTTGCTGCGTTGAAGTACTGGGGACCAGCCACCACCTTGAATTTCGCTGTAGAAGGGTACGAGAAGGAGATGGAGGAGATGAGTAAAGTGAGCAGAGAAGAGTACTTGGCTTCCCTCCGCCGCCGGAGCTCCGGCTTCTCCAGAGGCGTCTCTAAGTACCGCGGCGTCGCCAGGCATCACCATAATGGACGGTGGGAAGCGCGAATTGGAAGAGTGTTGGGGAACAAGTACCTCTACCTCGGCACTTTCAACACACAGGAGGAGGCGGCAGAGGCGTATGACATGGCGGCATTGGAATACAGAGGATCCAACGCTGTCACCAACTTCGACGTGGCCATTTACGTGGACCGCCTCAAGTCCAAAGGCGTCCAAGTCGACCAATTCCTCCAGCCAATTACTGACGTGGCAGCTGCTGAGGTGGCACCAGAACTATACATCAATGACGACAATGCAGCCAAATTAGAACCATCCTCTGCCTCGTCACCACTTCCCCTGCCCTCATCCTCATCCGAACAAGAGGTGGCAATGATGATGGCTATGCCTCTGCCACCGGAGATGCCTCCCACCACGACGACAACGAATAACGGTGGCAGCATGATGGAGCTTCTGGAGTTGGAGAATGATGAGAACTGGAGCTTCTGTTTCGAGTACCCAGAGGACAACGCGGCCTCCTCGCAGCTGGCCCCTGAGGAGGGGTGCTGTATCTTGCCGGAGTTGTTCGGCGTGGATGGGGGAGGGTTCCAGCTTGAGGACATTGACTACTTGGTGTTTGACTCGTACCCGACTACGGCGGCCGCGGATGATGATGACGTGGGGAAGAGCGTGAAGGAGAAGTTGTTGTTGTCGGAGGAGGATCATGGGGGCGGTGGGTCAAGGTCTCCTTCTTGTTCAACAACAACATCGGTTTCTTGTAACAAGGGTGGGCGCGCCGACCCAGCTTTCTTGTACAAAGTTGGCATTATAAGAAAGCATTGCTTATCAATTTGTTGCAACGAACAGGTCACTATCAGTCAAAATAAAATCATTATTTGCCATCCAGCTGATTCCCCCTAAAAGGATAACCCC

**Supplementary Figure 4.** Full-length cDNA sequence of *LuWRI1b*

**
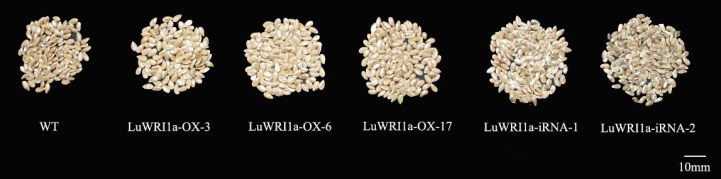
**

**Supplementary Figure 5**. Flax seed phenotype of WT and transgenic lines

**Supplementary Table 1.** Primers used for real time-PCR analysis

| **Gene** | **Forward Primer Sequence** | **Reverse Primer Sequence** |
| --- | --- | --- |
| GAPDH  BCCP2  FATA1  FUS3  LEC1  LEC2  L1L  ABI3  PDAT1  DGAT2 | 5'-AGGTTCTTCCCGCTCTCAAT-3'  5'- GGACCTCGTCAAGCTAGTGG-3'  5'- ACTGCTCGGATGCACATTGA-3'  5'- GAGGAGACCTCCATCCACCT -3'  5'- TCTCCCGGCAATGAATCCAC -3'  5'- GAGGAAGAGTGACGTTGGGG-3'  5'- CGCCAAGGAGACGATACAGG-3'  5'- GAGGAGACCTCCTTCCACCT -3'  5'- CGCATGACTCGTACATGGGA -3'  5'- ATATGGCTGGGGGCTATCCA -3' | 5'-CCTCCTTGATAGCAGCCTTG-3'  5'- TCATGACGGGAGGAGGAGTT-3'  5'- TTCACTTTGGCACCACGTCT -3'  5'- GGATCAATTTCACGTGCGGG -3'  5'- TGGCTATCGGCATGTACTGG -3'  5'- TTCGAGGACATACATCCGGC-3'  5'- GGGTGAGTGGCTCGATGTAG-3'  5'- GATCAATTTCACGTGCGGGG-3'  5'- AGGAGGACTCAAACCCCCTT -3'  5'- GCCCAAGTAGCTGGAATCGT -3' |
